# Depletions of Multi‐MeV Electrons and Their Association to Minima in Phase Space Density

**DOI:** 10.1029/2021GL097620

**Published:** 2022-04-18

**Authors:** A. Y. Drozdov, H. J. Allison, Y. Y. Shprits, M.E. Usanova, A. Saikin, D. Wang

**Affiliations:** ^1^ University of California Los Angeles Los Angeles CA USA; ^2^ GFZ German Centre for Geosciences Potsdam Germany; ^3^ Institute of Physics and Astronomy University of Potsdam Potsdam Germany; ^4^ Laboratory for Atmospheric and Space Physics University of Colorado Boulder Boulder CO USA

**Keywords:** radiation belts, EMIC, VERB, PSD

## Abstract

Fast‐localized electron loss, resulting from interactions with electromagnetic ion cyclotron (EMIC) waves, can produce deepening minima in phase space density (PSD) radial profiles. Here, we perform a statistical analysis of local PSD minima to quantify how readily these are associated with radiation belt depletions. The statistics of PSD minima observed over a year are compared to the Versatile Electron Radiation Belts (VERB) simulations, both including and excluding EMIC waves. The observed minima distribution can only be achieved in the simulation including EMIC waves, indicating their importance in the dynamics of the radiation belts. By analyzing electron flux depletions in conjunction with the observed PSD minima, we show that, in the heart of the outer radiation belt (L* < 5), on average, 53% of multi‐MeV electron depletions are associated with PSD minima, demonstrating that fast localized loss by interactions with EMIC waves are a common and crucial process for ultra‐relativistic electron populations.

## Key Points

- PSD minima are commonly observed throughout a year of observations, indicating a major role of fast‐localized losses of multi‐MeV electrons
- A modeled year reproduces the distribution of PSD minima over the same range of μ and K as observations only when EMIC waves are included
- On average, 53% of multi‐MeV flux depletions below L* = 5 (α_eq_ < 75 °) are associated with PSD minima

## 1 Introduction

Earth's outer radiation belt is a region of geomagnetically confined electrons and can contain particles with multi‐MeV energies. Changes in the flux of the multi‐MeV populations may be reversible (i.e., adiabatic) or irreversible and can arise from a number of processes, including large‐scale magnetic field fluctuations and wave–particle interactions. Analysis of phase space density (PSD) profiles (e.g., Green & Kivelson, 2004; Loridan et al., 2019; Selesnick & Blake, 2000; Shprits et al., 2017; Tu et al., 2009; Turner et al., 2010) is a commonly used tool in radiation belt research to determine the relative contributions of population changes due to ULF activity (monotonic radial profiles) and local acceleration (growing peaks profiles; Allison & Shprits, 2020; Baker, Jaynes, Li, et al., 2014; Chen et al., 2006; Iles et al., 2006; Olifer, Mann, Ozeke, Morley, & Louis, 2021; Reeves et al., 2013; Wu et al., 2020; Zhao et al., 2019). Shprits et al. (2017) noted that local deepening minima occurring in PSD profiles can also be indicative of fast localized loss processes, such as those resulting from resonant interactions with electromagnetic ion cyclotron (EMIC) waves studied here (e.g., Aseev et al., 2017; Blum et al., 2020; Capannolo, Li, Ma, Chen, et al., 2019; Kim et al., 2021; Ma et al., 2020; Xiang et al., 2017).

EMIC waves are highly efficient at scattering multi‐MeV electrons, and can play a major role in the occurrence of rapid depletions in the heart of the radiation belts (Shprits et al., 2013, 2016, 2018; Qin et al., 2019; Ukhorskiy et al., 2010; Xiang et al., 2017). At the outer boundary of the electron radiation belt, electrons can also be rapidly depleted by the combination of magnetopause shadowing and outward radial diffusion (Elkington et al., 2003; Hudson et al., 2014; Tu et al., 2019; Turner et al., 2012; Ukhorskiy et al., 2009; Xiang et al., 2017). ∼36% of geomagnetic storms result in a depletion of multi‐MeV electrons, while for lower energies, this percentage is significantly lower, suggesting energy‐dependent loss (Drozdov et al., 2019; Turner et al., 2019). Although studies have shown simultaneous observations of electron depletions below 1 MeV and EMIC wave activity (e.g., Capannolo, Li, Ma, Shen, et al., 2019), EMIC waves typically only resonate with higher energy electrons (Cao et al., 2017; Kersten et al., 2014; Mourenas et al., 2016; Zhang et al., 2016), with a minimum resonant energy commonly reported at ∼2 MeV. Whether EMIC waves affect lower energy electrons remains an open question (e.g., Ripoll et al., 2020). Despite observed EMIC waves often being very radially localized (Matsuda et al., 2021; Usanova et al., 2008, 2016; Usanova & Mann, 2016) and transient (e.g., Blum et al., 2016, 2017, 2021; Wang et al., 2017), studies have explored their importance for local multi‐MeV electron loss (e.g., Cervantes, Shprits, Aseev, Drozdov, et al., 2020; Drozdov et al., 2017, 2015; Xiang et al., 2018), however, the overall impact of EMIC waves on radiation belts as a whole is a subject of ongoing research (e.g., Ripoll et al., 2020). The consequences of EMIC wave activity to the overall shape and dynamics of the radiation belts still remains unknown.

To address the open questions raised above, here we perform analysis of observed PSD profiles to identify when fast localized loss occurs and explore the effect of EMIC waves. In this study, PSD minima are automatically identified using 1 year of Van Allen Probes and GOES observations (1 October 2012–1 October 2013). Dropouts in the electron fluxes occurring in conjunction with PSD minima are identified. We consider whether the affected energy range of where PSD minima are typically observed is consistent with the current theory of EMIC wave–particle interactions. Long‐term simulations with the VERB model are performed, including and excluding EMIC wave activity and minima in the modeled PSD profiles that are identified. We determined the values of first and second adiabatic invariants (μ and K) for which minima are found, and compare them to the corresponding values from observations. How frequently the PSD minima corresponds to the multi‐MeV electron flux depletion helps us to determine the significance of the fast‐localized losses caused by EMIC waves in the dynamics of the radiation belts.

## 2 Data

For the electron flux and PSD analysis in this work, we use Van Allen Probes (Spence et al., 2013) and Geostationary Operational Environmental Satellite (GOES) observations (e.g., Meredith et al., 2015; Rodriguez et al., 2014). The Van Allen Probes are two identical spacecraft (A and B) orbiting at low inclination (less than 18°) between ∼1.5 and ∼6 Earth radii, while GOES satellites operate at geostationary orbit. In this study, we use the GOES‐15 satellite data from the Energetic Proton, Electron, and Alpha particle Detector (EPEAD; energies >800 keV and >2 MeV) and the Magnetospheric Electron Detector (MAGED: energies ∼30–∼600 keV). Electron flux measurements from the Magnetic Electron Ion Spectrometer (MagEIS; energies ∼30 keV up to ∼4 MeV; Blake et al., 2013) and the Relativistic Electron Proton Telescope (REPT; energies ∼2–10 MeV; Baker et al., 2013) are used from the Van Allen Probes. Baker, Zhao, et al. (2019) showed that GOES‐15 and Van Allen Probes flux measurements showed good agreement when spacecraft were physically close, and hence, we omit extra intercalibration between the datasets. We calculate PSD using a 5‐min averaged flux measurement. The adiabatic invariants, equatorial pitch angle, and L* were calculated using the TS05 magnetic field model (Tsyganenko & Sitnov, 2005) and the International Geomagnetic Reference Field (IGRF) internal magnetic field model.

## 3 Methodology

To perform the search for PSD minima, we use automatic identification of the local minima and maxima along the profile. Extrema were identified numerically. The results were validated against manually found PSD minima from Aseev et al. (2017). Note that Aseev et al. (2017) used fewer than 2 months of observations, three values of the first adiabatic invariant, μ, and one value of the second adiabatic invariant, K. In our investigation, we extended the period of study (1 October 2012–1 October 2013) and the range of μ and K, as detailed below.

### 3.1 Data Preparation

The Van Allen Probes and GOES data are processed to obtain PSD in the extended range of μ ∈ [1,000, 5,500] MeV/G (10 values, distributed linearly with the step of 500 MeV/G), and K ∈ [0.001, 1] G^1∕2^R_E_ (10 values, with variable steps, e.g., K = 0.001, 0.003, 0.007, 0.01…1 G^1∕2^R_E_, see Figure 3). To obtain selected values of μ and K, we used interpolation, excluding points outside of the interpolation interval and measurements that were lower than the background level determined similarly to Shprits et al. (2018). PSD is binned into steps of 0.1 L* along each orbital pass (separating inbound and outbound passes) of Van Allen Probes A and B, taking the median within each bin, and extended the coverage at high L* using GOES observations.

As the search for the PSD local minima depends only on the shape of the profile, we normalized PSD profiles according to Equation 1:

(1) $$Norm.\;PSD=\frac{PSD\left(L^{*}\right)}{\int_{L^{*}=3.5}^{L^{*=5.5}}PSD\left(L^{*}\right)dL^{*}}$$

We search for local minima in the range L* ∈ [3.5, 5.5], as multi‐MeV electrons do not penetrate into the low L‐region (see Baker, Jaynes, Hoxie, et al., 2014).

### 3.2 Determining the PSD Minima Search Method

**Figure 1:**
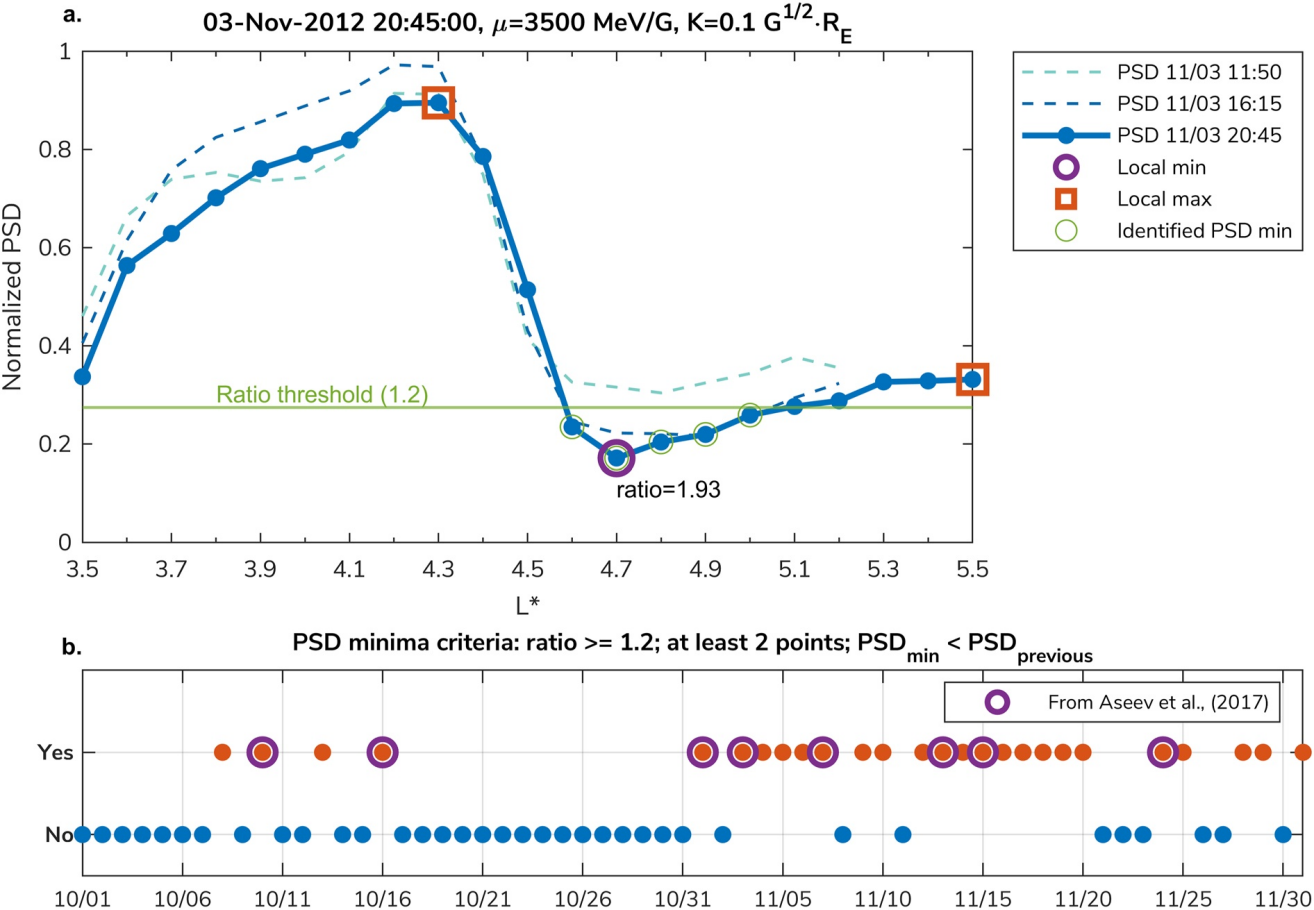
(a) Example of processed and normalized PSD profile (blue line). Purple and red markers show a local minimum and two local maxima. Green line is the ratio threshold below which the PSD minima are identified (green markers). Dashed profiles correspond to the PSD at the previously available pass, showing that the identified minimum is deepening. (b) Formation of the PSD minima between 9 October and 29 November 2012. Red points ‐ automatically identified PSD minima (at least one per day). Blue points ‐ days without formation of PSD minima. Purple circles ‐ PSD minima identified by Aseev et al. (2017).

Figure 1a shows an example of a processed PSD profile. To determine appropriate criteria for classifying local minima, we calculate the ratio between the local minimum and the smallest adjacent local maximum. In Figure 1a, the smallest local maximum is located at L* = 5.5, at the edge of the PSD profile, and the resulting ratio is 1.93. A minimum is then identified if this ratio is larger than the ratio threshold (green line in Figure 1a). Additionally, to exclude small variations that can result in a single point local minimum, we require that at least two points are below the ratio threshold. Hence, the narrowest localized PSD minimum that can be defined by this algorithm is 0.2 L* wide. In this study, as we focus on the effect of localized losses, we employ an additional criterion that PSD at the local minimum must be lower than for the previously available satellite pass. This criterion ensures that we only detect deepening minima.

After establishing the identification criteria, we select the ratio threshold based on PSD minima that were manually identified by Aseev et al. (2017) in an interval between 9 October and 29 November 2012, which showed multiple events of enhanced EMIC wave activity observed on the ground (Usanova et al., 2014). In their study, PSD profiles were analyzed for μ = 2,500, 3,500, 4,500 MeV/G at K = 0.1 G^1∕2^R_E_, and eight events of pronounced minima were found. Figure 1b shows the number of days when PSD minima are detected using our algorithm in comparison to the events manually found by Aseev et al. (2017). We successfully identify the previously detected PSD minima when the ratio threshold is set to ≥1.2. Increasing the threshold led to not identifying all minima from the Aseev et al. (2017) study. Our algorithm identified more events than Aseev et al. (2017), because the authors only focused on the first appearance of the pronounced PSD minima. Several identified PSD minima are persistent and continued to decrease after the initial appearance. In Section 4, we compare the identified PSD minima events throughout the entire 1‐year period to associated electron flux depletions to identify the PSD minima driven by localized loss.

Using our algorithm, we perform the search of PSD minima for the 1‐year period and, following Aseev et al. (2017), the found minima are grouped daily. To confirm that these PSD minima are a result of EMIC wave activity, we perform a numerical simulation using the Versatile Electron Radiation Belts (VERB) code.

### 3.3 The VERB Code Simulations

In order to perform long‐term simulations (from 1 October 2012 to 1 October 2013) with and without EMIC waves, we use a similar model setup as in Drozdov et al. (2017) (see Text S1 in Supporting Information S1). The VERB code solves the Fokker‐Planck equation using an approach of a single grid of modified adiabatic invariants (Subbotin & Shprits, 2012). The simulation includes Kp‐driven hiss (Spasojevic et al., 2015) and chorus (Zhu et al., 2019) waves; constant lightning‐generated whistler waves and very low frequency (VLF) waves from man‐made transmitters (Subbotin et al., 2011). When enabled, EMIC waves (Meredith et al., 2014) are parameterized by solar wind dynamic pressure according to Drozdov et al. (2017) (see Figure S1 in Supporting Information S1). The plasmapause location is defined by Carpenter and Anderson (1992). We use the Kp‐dependent electromagnetic part of Brautigam and Albert (2000) radial diffusion parameterization, which is consistent with our previous simulations, and provide optimal performance based on the comparative analysis among other radial diffusion parameterizations (see Drozdov et al., 2021). The initial and outer boundary (L* = 5.5) conditions are set by Van Allen Probes measurements. Other boundary conditions and the size of the simulation domain are the same as in Drozdov et al. (2017), and the simulation timestep is set to 1 hour.

### 3.4 Searching for Multi‐MeV Electron Flux Depletion Events

Identifying multi‐MeV electron flux depletions allows us to associate PSD minima with observed electron loss. In order to find periods when the multi‐MeV electrons show a net depletion, we bin the electron flux with a time step of 8 hr, and an L* step of 0.5. Then we perform a moving median analysis with a time window of 24 hr and calculate the difference between the logarithm of fluxes at the selected time and 24 hr later. We defined a flux depletion event if the flux decreases by a factor of 3 within the 24 hr period according to Equation 2:

(2) $$\Delta log_{10}\left(j\right)=log_{10}\left(\bar{j_{t_{0}\text{…}t_{0}+24h}}\right)-log_{10}\left(j_{t_{0}}\right)\leq log_{10}\left(1/3\right)$$

We search for flux depletion events in nine energy channels using the REPT instrument (E ∈ [1.8, 9.9] MeV), at 6 equatorial pitch angles ($\alpha_{eq}=15^{{}^{\circ}},\;30^{{}^{\circ}},45^{{}^{\circ}},60^{{}^{\circ}},75^{{}^{\circ}},85^{{}^{\circ}}$), and at 7 L* values (L* ∈ [3, 6], ΔL* = 0.5). Note that we interpolate flux to obtain values at fixed equatorial pitch angles.

**Figure 2:**
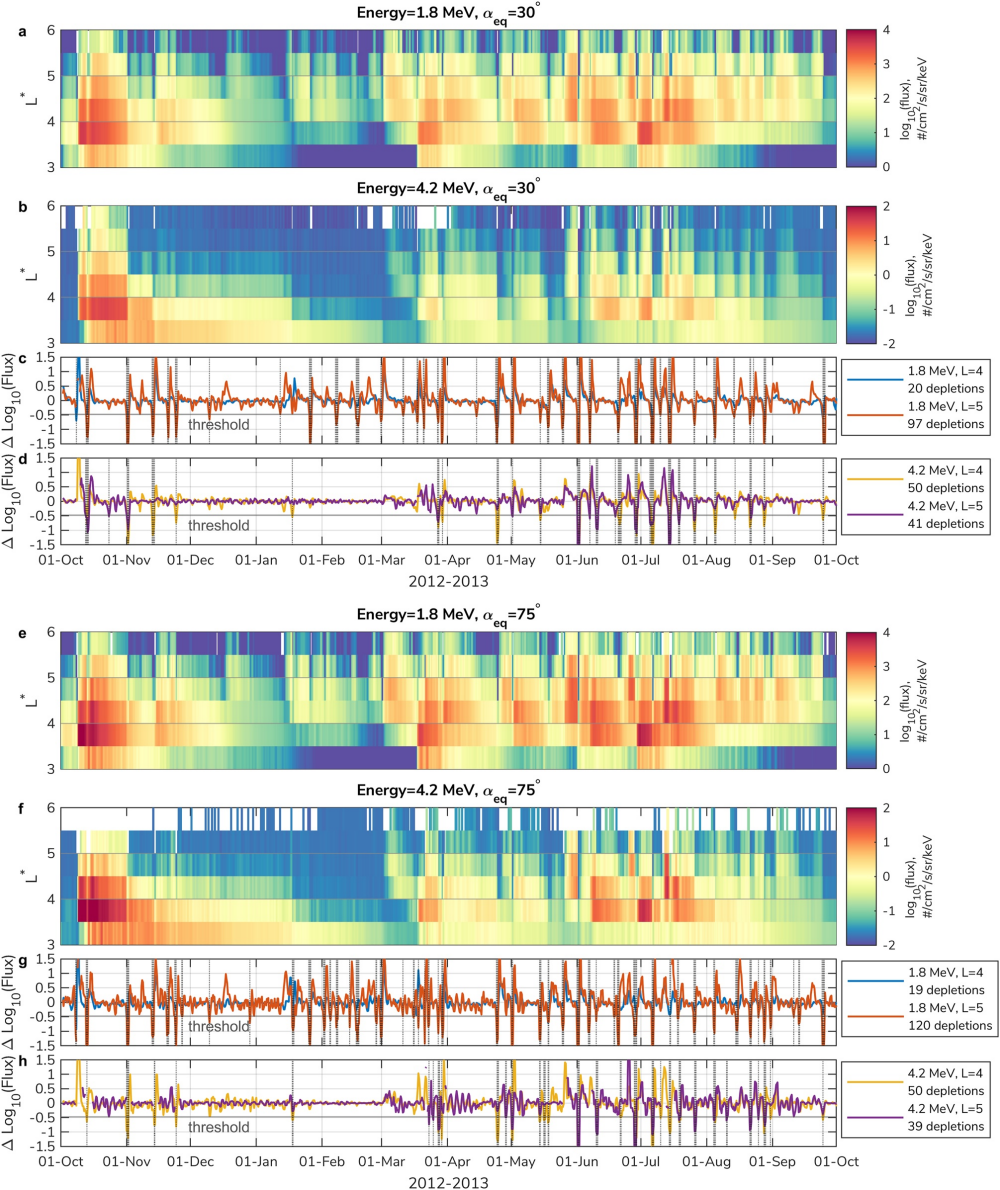
Observed electron flux at (a)–(d) $\alpha_{eq}=30^{{}^{\circ}}$, (e)–(h) $\alpha_{eq}=75^{{}^{\circ}}$; at (a), (e) 1.8 and (b), (f) 4.2 MeV. (c, d, g, h) $\Delta log_{10}\left(j\right)$constructed at L* = 4 and L* = 5, at 1.8 and 4.2 MeV. Vertical dotted lines correspond to the found flux depletion events ($\Delta log_{10}\;\left(j\right)\leq log_{10}\;\left(1/3\right)$).

Figures 2a and 2b show an example of 1.8 and 4.2 MeV electron flux at $\alpha_{eq}=30{\;}^{{}^{\circ}}$and $\alpha_{eq}=75{\;}^{{}^{\circ}}$during the period of interest. Figures 2c–2g and  2h show the calculated difference of logarithm of flux according to Equation 2. Horizontal solid lines indicate the thresholds of the electron flux depletion and when the differences cross the threshold, an electron flux depletion event is identified (vertical dotted lines). There are significantly more depletion events of 1.8 MeV electrons at $L^{*}=5$(97–120) than at $L^{*}=4$(∼20). For 4.2 MeV electrons, the number of depletions is similar at $L^{*}=4$and $L^{*}=5$(∼40–50). Enhancements of 1.8 MeV electrons are more common, hence depletions are observed more often. However, 4.2 MeV electron enhancements are less common (e.g., Baker, Hoxie et al., 2019), but EMIC waves are more effective at scattering electrons at those energies.

## 4 Results

### 4.1 Distribution of PSD Minima

**Figure 3:**
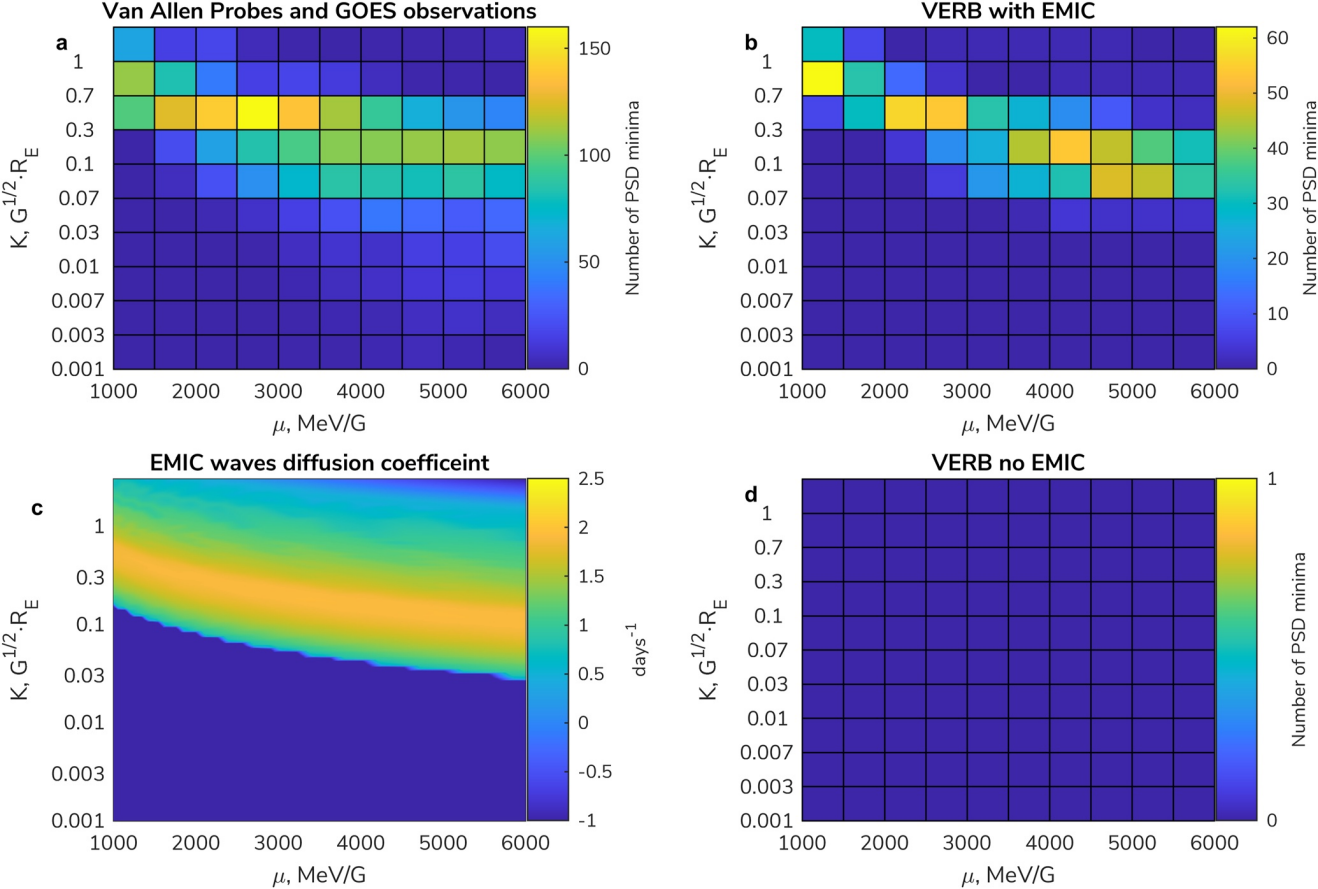
Distribution of the PSD minima in μ and K space. Each cell corresponds to the number of days when PSD minima were detected. The distribution is constructed based on: (a) Van Allen Probe and GOES observations, VERB code simulation (b) with and (c) without EMIC waves. (c) EMIC wave pitch‐angle diffusion coefficient at L = 4.

Figure 3a shows the distribution across μ and K, where deepening PSD minima are observed by Van Allen Probes and GOES. The statistics show that PSD minima are seen regularly, observed for hundreds of days in particular cells, over the year period. We see that for lower values of μ, PSD minima are seen most commonly at the highest values of K, and as μ increases, the most common K value for the deepening minima decreases. Figure 3b shows a similar histogram as in Figure 3a, with PSD minima identified from the output of the VERB simulation with EMIC waves. The distribution of PSD depletions across μ and K space very closely resembles the distribution obtained from the observations, both in terms of the typical μ and K coverage and the trends seen. This agreement suggests that the parameterized model of the EMIC wave diffusion coefficients used in the simulation well covers the typical μ and K values where the effects of EMIC waves are generally seen. A quantitative difference is, however, observed, which can be explained by the idealistic nature of the simulation results. The depletion of the PSD due to the EMIC waves strictly obeys the diffusion process, while observations include small variations from orbit to orbit. This leads to some over‐counting of the persistent minima that are present after the electron flux depletion. The automatic algorithm periodically detects PSD at the minimum that is lower than on the previously available orbit, and therefore, counts it as an event, while the simulation results provide a stable and smooth PSD change and count only the individual occurrences of the flux depletion. Applying harder criteria to the algorithm to counter this effect led to the disappearance of the events found by Aseev et al. (2017). Nevertheless, the results of the simulation with EMIC waves confirm the dynamics of multi‐MeV electrons fluxes, as shown by Drozdov et al. (2017).

The VERB simulation, which does not include EMIC waves, results in a total absence of deepening PSD minima (Figure 3d). Therefore, all PSD minima in the simulation with EMIC waves are a result of EMIC wave activity. Figure 3c shows pitch‐angle diffusion coefficients in μ and K space due to EMIC waves that are used in the simulation. Once again, there is a close resemblance between the distribution of the EMIC wave pitch‐angle diffusion coefficient (Figure 3c) and where the deepening PSD minima were most readily observed in data (Figure 3a), suggesting that the quasi‐linear theory for the energy range over which EMIC waves are resonant is well supported by the occurrence distribution of PSD minima observations. Additionally, the agreement between the observed and the modeled minima distributions shown in Figures 3a and 3b indicates that the observed PSD minima in Figure 3a are likely the result of EMIC activity.

### 4.2 Conformity Between PSD Minima and Electron Flux Depletions

**Figure 4:**
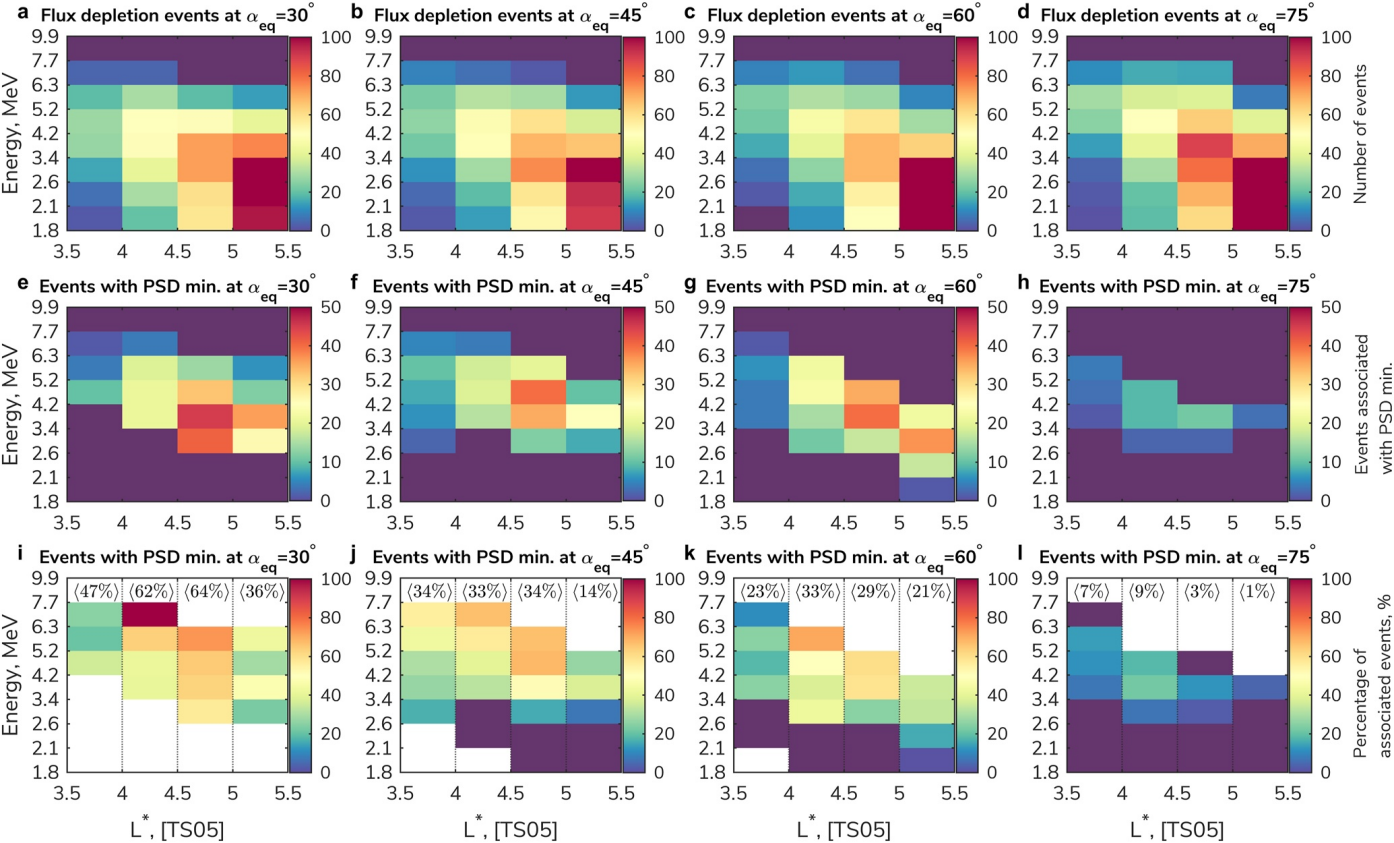
(a, b, c, d) Distribution of the electron flux depletion events in L* and energy space at different equatorial pitch angles (from left to right column: $\alpha_{eq}=\;30^{{}^{\circ}},45^{{}^{\circ}},60^{{}^{\circ}},75^{{}^{\circ}}$, respectively). (e, f, g, h) Distribution of the electron flux depletion events occurring in conjunction with minima in PSD. (i, j, k, l) The percentage of the flux depletion events that are associated with PSD minima in comparison to all flux depletion events (average per L* is shown on top). Deep purple color corresponds to zero. White color corresponds to absence of the data.

To ascertain the role EMIC wave‐driven loss plays in the overall depletions in radiation belt flux, the depletion events identified in Section 3.4 are compared with the observed PSD minima. The first row in Figure 4 (a, b, c, and d) shows the statistics of all found flux depletions across different energies and L* for four values of equatorial pitch angle. No depletions are recorded at energies >7.7 MeV (deep purple color), simply because there is no electron flux above the background noise level. A noticeably large number of electron flux depletions occurred at L* > 5, with a distribution similar for different pitch angles, consistent with frequently occurring outward radial diffusion and magnetopause shadowing (e.g., Drozdov et al., 2019; Fei et al., 2006; Olifer, Mann, Ozeke, Claudepierre, et al., 2021; Pierrard et al., 2020, 2021; Xiang et al., 2017). The number of depletion events at L* > 5 decreases with increasing energy, because high‐energy electrons occur less frequently in the radiation belts.

In order to compare the electron flux depletion events with the occurrence of PSD minima, we convert the energies and pitch angles at which depletions were identified to μ and K using a dipole magnetic field model and then search for PSD minima within ±12 hr. The second row in Figure 4 (e, f, g, and h) shows electron flux depletion events that are associated with PSD minima via this technique. There are significantly fewer depletion events at high equatorial pitch angle ($\alpha_{eq}=75^{{}^{\circ}}$) in comparison with lower pitch angles ($\alpha_{eq}<75^{{}^{\circ}}$) when the associated PSD minima restriction is applied. This is consistent with diffusion by EMIC waves since, alone, they do not typically affect electrons with equatorial pitch angles near 90°, but are very effective at lower pitch angles (e.g., Jordanova et al., 2008; Kersten et al., 2014; Shprits et al., 2016; Usanova et al., 2014).

The last row in Figure 4 (i, j, k, and l) shows the percentage of all the depletion events in panels a–d that are associated with local deepening minima in PSD. Note that μ=1,000MeV/G corresponds to energy of ∼1–4 MeV (depending on $L^{*}$ at $\alpha_{eq}=30^{{}^{\circ}}-75^{{}^{\circ}}$) and μ=5,500MeV/Gcorresponds to energy of ∼4–10 MeV. Excluding bins outside of the valid μand Krange, we find that the percentage of depletion events associated with local deepening minima is dependent on the equatorial pitch angle. For the lowest equatorial pitch angle bin, on average, 53% of the depletion events for L* < 5 have an associated minimum in the PSD profiles (47%–64% per L* bin), suggesting that EMIC wave scattering is partly responsible for the identified flux depletions at this pitch angle, despite the localized and transient nature of EMIC waves. At higher pitch angles, the percentage of depletions associated with PSD mimina decreases, showing 33%–34% for the 45° equatorial pitch angle bin; and 23%–33% at 60° per L* bin. Depletion events associated with PSD minima are very infrequent at the highest equatorial pitch angle considered (only 6% on average at L* < 5 at $\alpha_{eq}=75^{{}^{\circ}}$, 3%–9% per L* bin, Figure 4l). The small percentage here is likely a result of the combination of pitch‐angle scattering by hiss and chorus waves with EMIC waves (e.g., Drozdov et al., 2020). Figure 4 also shows that depletions with associated PSD minima are rarely observed at L* > 5 (1%–21%, on average, at $\alpha_{eq}\geq 45^{{}^{\circ}}$, panels j–l) and, as such, the flux depletions observed in this L* range are likely to be primarily due to magnetopause shadowing (which is more effective with increasing pitch angles) and associated effects.

The large percentage of depletion events that are associated with PSD minima indicates that, very often, the depletion of the multi‐MeV electron radiation belts is linked to fast localized loss processes originating from EMIC wave activity, consistent with previous results (e.g., Cervantes, Shprits, Aseev, & Allison, 2020; Ross et al., 2021; Xiang et al., 2018).

## 5 Conclusions

In this study, we performed a statistical analysis of the occurrence of deepening PSD minima and explored their relation to multi‐MeV flux depletions. We find that deepening minima in PSD, which can only be formed by a fast localized loss process, are commonly observed in the outer radiation belt (L* between 3.5 and 5.5) at multi‐MeV energies. The observed distribution across μ and K space of the PSD minima shows close agreement with both the values of EMIC wave diffusion coefficients as well as the distribution of PSD minima achieved in the VERB simulation with EMIC waves included. When EMIC wave activity was omitted from the simulation, deepening PSD minima were not identified. We therefore conclude that EMIC waves play a significant role in the formation of PSD minima in the radiation belts and that the quasi‐linear theory for the electron energy range affected by EMIC waves is well supported by the observed occurrence distribution of PSD minima.

Electron flux depletion events were most frequently identified at L* > 5 but, of these, only 1%–21% ($\alpha_{eq}\geq 45^{{}^{\circ}}$) had associated PSD minima. We consider these depletions to be a result of outward radial diffusion and magnetopause shadowing. Depletion events, which were associated with PSD minima, were rarely observed at high equatorial pitch angle ($\alpha_{eq}=75$), indicating the dominating role of other loss processes, aside from EMIC waves in this pitch angle range (despite possible EMIC wave scattering at lower pitch angle). For L* < 5, simultaneous observations of multi‐MeV electron flux depletions and PSD minima occurred often (from 23% to 64%, on average, depending on pitch angle and L*). The large percentage of electron flux depletion events which were observed in conjunction with PSD minima profiles indicates the significant role of fast localized loss processes in the dynamics of the multi‐MeV radiation belts. In future research, we will investigate a longer period and extended energy range (including sub‐MeV electrons).

## Supporting information

Supporting Information S1

## Data Availability

We thank the Van Allen Probe ECT team for providing the data (https://rbsp-ect.newmexicoconsortium.org/). The GOES measurements are available at the NOAA NGDC website (https://ngdc.noaa.gov/stp/satellite/goes/dataaccess.html). The authors used geomagnetic indices provided by OMNIWeb (https://omniweb.gsfc.nasa.gov/). The data to reproduce the figures are available at UCLA dataverse repository (https://doi.org/10.25346/S6/B05T1T).
