# Supplementary material for: Depletions of Multi‐MeV Electrons and Their Association to Minima in Phase Space Density
Source: Geophys Res Lett. 2022 Apr 18;49(8):e2021GL097620. doi: 10.1029/2021GL097620 (PMC9286695; doi:10.1029/2021GL097620)
Supplement: Supplementary file 1 — Supporting Information S1 [file GRL-49-0-s001.pdf]

AGU PUBLICATIONS

Geophysical Research Letters

Supporting Information for

Depletions of Multi-MeV Electrons and Their Association to Minima in Phase Space Density

A.Y. Drozdov[1], H.J. Allison[2], Y.Y. Shprits[1, 2, 3], M. Usanova[4], A. Saikin[1], D. Wang[2]

[1] University of California Los Angeles, CA, USA
[2] GFZ German Centre for Geosciences, Potsdam, Germany
[3] Institute of Physics and Astronomy, University of Potsdam, Germany
[4] Laboratory for Atmospheric and Space Physics, University of Colorado Boulder, Boulder, Colorado, USA

Contents of this file

Text S1
Figure S1

Introduction

This document includes a description of the Versatile Electron Radiation Belt (VERB) code, simulation setup, and the figure showing a comparison of the modeled and observed electron flux.

Text S1. The VERB code

The Versatile Electron Radiation Belt (VERB) code (*Subbotin and Shprits*, 2009) is designed to model the radiation belt dynamics by solving the Fokker-Planck equation numerically. Subbotin and Shprits (2012) suggested performing 3D simulations on a single grid of modified adiabatic invariants. This approach is used in this study and allows the elimination of interpolation between the numerical grids, which can either lead to numerical errors or, in the case of spline interpolation, unstable behavior of the code. In this formulation, the Fokker-Planck equation can be written as:

$$\frac{\partial f}{\partial t} = \frac{1}{G}\frac{\partial}{\partial L^*}\bigg|_{V,K} G\langle D_{L^*L^*}\rangle \frac{\partial f}{\partial L^*}\bigg|_{V,K} + \frac{1}{G}\frac{\partial}{\partial V}\bigg|_{L^*,K} G\left(\langle D_{VV}\rangle \frac{\partial f}{\partial V}\bigg|_{L^*,K} + \langle D_{VK}\rangle \frac{\partial f}{\partial K}\bigg|_{L^*,K}\right) + \frac{1}{G}\frac{\partial}{\partial K}\bigg|_{L^*,V} G\left(\langle D_{KK}\rangle \frac{\partial f}{\partial K}\bigg|_{L^*,V} + \langle D_{VK}\rangle \frac{\partial f}{\partial V}\bigg|_{L^*,V}\right) - \frac{f}{\tau} \quad (1)$$

where f is the three-dimensional PSD, V and K are the modified adiabatic invariants, $V \equiv \mu \cdot (K + 0.5)^2$, $K \equiv J/\sqrt{8m_0\mu}$, μ is first adiabatic invariant, m_0 is the electron mass, $\langle D_{L^*L^*}\rangle$ are radial diffusion coefficients, $\langle D_{VV}\rangle$, $\langle D_{KK}\rangle$ and $\langle D_{VK}\rangle$ are the bounce-averaged diffusion coefficients, $G = -2\pi B_0 R_E^2 {L^*}^{-2}\sqrt{8\mu m_0}/(K + 0.5)^2$ is the Jacobian of the transformation from an adiabatic invariant system (μ, J, Φ), R_E is the Earth's radius, $B_0 = 0.3$ G is the field on the equator at the Earth's surface, c is the speed of light, and f/τ is the loss term, where τ represents the electron's lifetime inside the loss cone and is equal to a quarter of the bounce period. V and K are convenient for numerical calculations because K is independent of the particle's energy, and V depends weakly on the particle's pitch angle.

The Kp-dependent radial diffusion coefficient adopted from Brautigam and Albert (2000) was previously used in the VERB code long-term simulations (e.g., *Subbotin et al.*, 2011; *Kim and Shprits*, 2013), and the model results agreed well with the observations of relativistic electron fluxes. Drozdov et al. (2021) showed VERB code simulations with the electromagnetic part of the parameterization by Brautigam and Albert (2000) providing optimal performance based on the comparative analysis, among other parameterizations.

The bounce- and MLT-averaged diffusion coefficients are computed using the Full Diffusion Code (*Shprits and Ni*, 2009). The simulation includes dayside and nightside chorus waves that produce diffusion outside the plasmasphere. Inside the plasmasphere, the simulations include hiss waves, lightning-generated whistler waves, and anthropogenically-generated very low frequency (VLF) waves. The chorus and the hiss wave frequency and amplitude statistical model is obtained based on the Van Allen Probes measurements (*Spasojevic et al.,* 2015; *Zhu et al.,* 2019). The wave spectrum and wave normal angle distributions are defined by gaussian function. The wave intensity is defined as a function of Kp-index. Wave frequency, intensity, and wave normal angle distribution for VLF plasma waves and lightning-generated whistlers are taken from Subbotin et al. (2011). The location of the plasmapause is calculated following Carpenter and Anderson (1992).

The diffusion coefficients for helium band EMIC waves are calculated with the spectral properties from Meredith et al. (2014). The spectrum is approximated by gaussian function. Central frequency, frequency bandwidth, and lower and upper cutoff frequencies are 3.6 f_{O+}, 0.25 f_{O+}, 3.35 f_{O+}, and 3.85 f_{O+}, where f_{O+} is the oxygen gyrofrequency. The coefficients are scaled according to these waves' magnetic local time (MLT) distribution (25%) and wave occurrence rate (2%), following Meredith et al. (2014). It should be noted that wave power B_w^2, MLT distribution, and wave occurrence rate are linear multipliers in the EMIC wave diffusion coefficients. The ion composition used in the diffusion coefficient computation is 70% H+, 20% He+, and 10% O+. According to Drozdov et al. (2017), EMIC waves are activated in the

simulations when the solar wind dynamic pressure is larger than or equal to 3 nPa. The EMIC wave power B_w^2 is scaled at 0.4 nT^2.

The computational orthogonal grid has 101x100x46 points for V, K and L^*, respectively. The L^* range varies from 1.0 to 5.5. The boundary conditions are set at L^*=5.5 for energies from 10 keV to 10 MeV and pitch angles from 0.7° to 89.3°, respectively. The V-grid points are distributed logarithmically, and the K and L^* grid-points are distributed linearly.

The initial conditions are obtained from the Van Allen Probes observations. The PSD for the lower V-boundary (10 keV at L^*=5.5 and adiabatically increasing at lower L-shells) is set to an initial value and remains constant, representing the balance between convective losses and sources. The PSD of upper V-boundary corresponds to 10 MeV at L^*=5.5 and adiabatically increases in energy towards lower L-shells, and its value is set to zero, representing the absence of very high-energy electrons. The lower K-boundary represents the loss cone, and PSD is set to zero. The upper K-boundary condition is set to a zero-gradient PSD, representing the flat distribution at 90° (*Horne et al.*, 2003). PSD at the lower radial boundary ($L^* = 1$) is set to zero and represents losses into the atmosphere. The PSD required for the upper radial boundary ($L^* = 5.5$) condition is updated at every step of the simulation and obtained from the Van Allen Probes observation.

References

Brautigam, D. H., and J. M. Albert (2000), Radial diffusion analysis of outer radiation belt electrons during the October 9, 1990, magnetic storm, *J. Geophys. Res.*, *105*(A1), 291–309, doi:10.1029/1999ja900344.

Carpenter, D. L., and R. R. Anderson (1992), An ISEE-whistler model of equatorial electron density in the magnetosphere, *J. Geophys. Res. [Space Phys]*, *97*(A2), 1097–1108, doi:10.1029/91JA01548.

Drozdov, A. Y., Shprits, Y. Y., Usanova, M. E., Aseev, N. A., Kellerman, A. C., & Zhu, H. (2017). EMIC wave parameterization in the long-term VERB code simulation. *Journal of Geophysical Research, [Space Physics]*, *122*(8), 2017JA024389. https://doi.org/10.1002/2017JA024389

Drozdov, A. Y., Allison, H. J., Shprits, Y. Y., Elkington, S. R., & Aseev, N. A. (2021). A comparison of radial diffusion coefficients in 1-D and 3-D long-term radiation belt simulations. *Journal of Geophysical Research, [Space Physics]*, *126*(8). https://doi.org/10.1029/2020ja028707

Horne, R. B., N. P. Meredith, R. M. Thorne, D. Heynderickx, R. H. A. Iles, and R. R. Anderson (2003), Evolution of energetic electron pitch angle distributions during storm time electron acceleration to megaelectronvolt energies, *J. Geophys. Res. [Space Phys]*, *108*(A1), SMP 11–1–SMP 11–13, doi:10.1029/2001JA009165

Kim, K.-C., and Y. Shprits (2013), Long-term relativistic radiation belt electron responses to GEM magnetic storms, *J. Atmos. Sol. Terr. Phys.*, *100–101*(0), 59–67, doi:10.1016/j.jastp.2013.04.007.

Ma, Q. et al. (2015), Modeling inward diffusion and slow decay of energetic electrons in the Earth's outer radiation belt, *Geophys. Res. Lett.*, *42*(4), 2014GL062977, doi:10.1002/2014GL062977.

Meredith, N. P., R. M. Thorne, R. B. Horne, D. Summers, B. J. Fraser, and R. R. Anderson (2003), Statistical analysis of relativistic electron energies for cyclotron resonance with EMIC waves observed on CRRES, *J. Geophys. Res.*, *108*(A6), 1250, doi:10.1029/2002JA009700.

Shprits, Y. Y., and B. Ni (2009), Dependence of the quasi-linear scattering rates on the wave normal distribution of chorus waves, *J. Geophys. Res. [Space Phys]*, *114*(A11), doi:10.1029/2009JA014223.

Spasojevic, M., Y. Y. Shprits, and K. Orlova (2015), Global empirical models of plasmaspheric hiss using Van Allen Probes, *J. Geophys. Res. [Space Phys]*, 120(12), 2015JA021803, doi:10.1002/2015JA021803

Subbotin, D. A., and Y. Y. Shprits (2009), Three-dimensional modeling of the radiation belts using the Versatile Electron Radiation Belt (VERB) code, *Space Weather*, *7*(10), S10001.

Subbotin, D. A., and Y. Y. Shprits (2012), Three-dimensional radiation belt simulations in terms of adiabatic invariants using a single numerical grid, *J. Geophys. Res.*, *117*(A5), A05205, doi:10.1029/2011JA017467.

Subbotin, D. A., Y. Y. Shprits, and B. Ni (2011), Long-term radiation belt simulation with the VERB 3-D code: Comparison with CRRES observations, *J. Geophys. Res. [Space Phys]*, *116*(A12), A12210, doi:10.1029/2011JA017019.

Zhu, H., Shprits, Y. Y., Spasojevic, M., & Drozdov, A. Y. (2019). New hiss and chorus waves diffusion coefficient parameterizations from the Van Allen Probes and their effect on long-term relativistic electron radiation-belt VERB simulations. *Journal of Atmospheric and Solar-Terrestrial Physics*, *193*, 105090. https://doi.org/10.1016/j.jastp.2019.105090

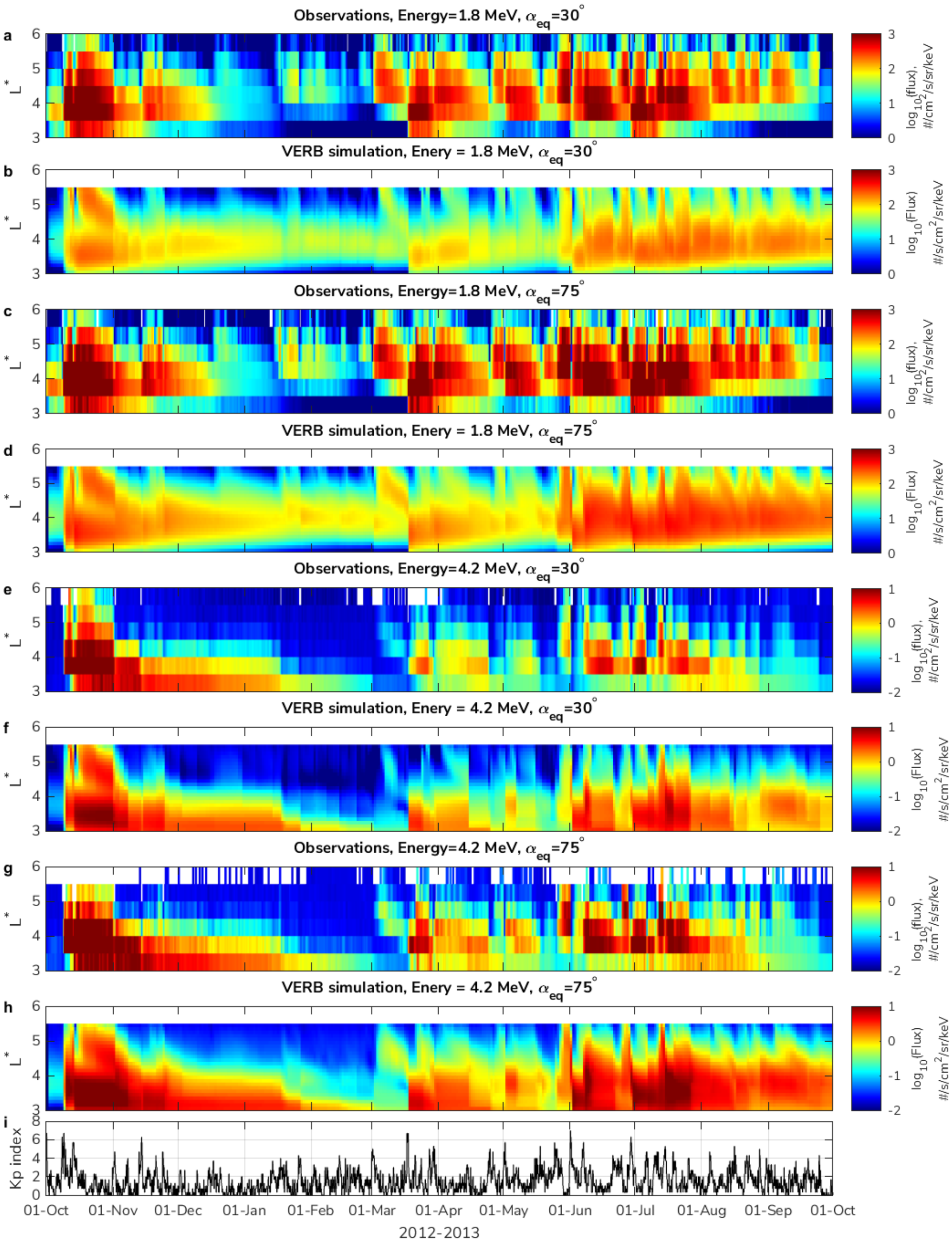

Figure S1. Evolution of the 1.8 and 4.2 MeV electron fluxes at the pitch angles of 30° and 75°. (a, c, e, g) Observations; (b, d, f, h) VERB code simulation with EMIC waves; i) Kp index.
